# Private practice dentists improve antibiotic use after dental antibiotic stewardship from infectious diseases experts

**DOI:** 10.1017/ash.2022.191

**Published:** 2022-05-16

**Authors:** Debra Goff, Julie Mangino, Elizabeth Trolli, Douglas Goff

## Abstract

**Background:** Dentists prescribe ~25.7 million antibiotic prescriptions annually. Private practice dentists (PPDs) represent 80% of US dentists who need to implement dental antimicrobial stewardship. We conducted a prospective cohort study of PPDs comparing appropriateness of antibiotic use before and after dental AS education. **Methods:** PPDs were invited to participate in this study. In phase 1 (pre-education), we collected 3 months (June–August 2019) of retrospective antibiotic use data (indication, dose, duration, penicillin allergy history) and number of dental procedures. We also conducted a preliminary survey to assess baseline antimicrobial stewardship knowledge. In phase 2 (education), PPDs attended 4 televideo education sessions (March–May 2021) taught by an infectious disease– antimicrobial stewardship (ID-AS) pharmacist and physician. In phase 3 (posteducation), we prospectively collected 3 months (June–August 2021) of antibiotic use data (as in phase 1), using an online database with ongoing feedback. In phase 4, we conducted antibiotic use audit and feedback to PPDs after the survey, and we solicited recommendations to reach more PPDs. The Student *t* test was used for statistical analyses. **Results:** Study participants comprised 15 PPDs: 2 oral maxillofacial surgeons, 6 periodontists, 4 endodontists, and 3 general dentists. Among them, 10 had been in practice >20 years. The presurvey revealed that 14 were unfamiliar with dental antimicrobial stewardship. All prescribed clindamycin (25% for nonpenicillin allergy), and standard antibiotic duration ranged from 5 to 14 days based on dental school training. In phase 3, despite more procedures, overall antibiotic use and duration decreased, and the use of clindamycin, quinolones, and prophylaxis for joint implant patients, also decreased. Appropriate use improved from 22% to 95%. Postsurvey responses on perceived value of antimicrobial stewardship education were 100% positive, with recommendations to make antimicrobial stewardship a required annual continuing education, similar to opioid continuing education. Study participants invited the ID-AS experts to teach an additional 150 PPDs to date via established PPD study clubs to expand dental antimicrobial stewardship across the United States. **Conclusions:** After learning dental antimicrobial stewardship guidance from ID-AS experts, PPDs rapidly optimized antibiotic prescribing behavior. PPDs identified their established study clubs as a forum to quickly expand dental antimicrobial stewardship training by ID-AS experts throughout the United States.

**Funding:** MERCK

**Disclosures:** None

